# Lumbar Disc Degeneration Is Linked to Dorsal Subcutaneous Fat Thickness at the L1–L2 Intervertebral Disc Level Measured by MRI

**DOI:** 10.3390/tomography10010012

**Published:** 2024-01-17

**Authors:** Ibrahem Hussain Kanbayti, Abdulrahman S. Al-Buqami, Mohammad H. Alsheikh, Saad M. Al-Malki, Ibrahim Hadadi, Adnan Alahmadi, Bander S. Almutairi, Hamzah H. Ahmed

**Affiliations:** 1 Radiologic Sciences Department, Faculty of Applied Medical Sciences, King Abdulaziz University, Jeddah, Saudi Arabia; abdulrahman.safar91@gmail.com (A.S.A.-B.); salmalky1234@gmail.com (S.M.A.-M.); aaalahmadi@kau.edu.sa (A.A.); hhahmed@kau.edu.sa (H.H.A.); 2 Department of Radiological Sciences, College of Applied Medical Sciences, King Khalid University, Abha, Saudi Arabia; ihadadi@kku.edu.sa; 3 Department of Radiology, King Abdulaziz University Hospital, Jeddah, Saudi Arabia; balmotairi@kau.edu.sa

**Keywords:** degenerative disc disease, dorsal subcutaneous fat, magnetic resonance imaging, obesity, intervertebral disc

## Abstract

Background: Obese individuals have a higher risk of degenerative disc disease (DDD). Currently, body mass index is not sensitive enough to differentiate between muscle and fat distribution, and obesity-related health issues are linked to the way body fat is distributed. Therefore, this study aims to investigate the association between the dorsal subcutaneous fat thickness (DSFT) of the lumbar spine, an alternative measurement tool of body fat distribution, and DDD. Methods: A total of 301 patients with DDD and 123 participants without the disease were recruited. Using length functions of magnetic resonance imaging (MRI) console, the DSFT of L1 to S1 intervertebral disc levels was measured in mid-sagittal spin-echo T2 weighted image. The Mann–Whitney U test and Chi-squared test (X2) were utilized to examine any variations between the case and control groups. Logistic regression models were built to explore the association of the DSFT with DDD. Results: The logistical regression model showed a positive association between DDD and DSFT [OR: 1.30, 95% CI: 1.02–1.64, *p =* 0.03]. In the stratified logistic regression analysis, a positive association was found between DDD and DSFT among younger participants and females [OR _young_: 1.48; 95% CI (1.02–2.20); *p =* 0.04—OR _female_: 1.37; 95% CI (1–1.88); *p =* 0.05]. Conclusions: Younger females with thicker DSFT at the L1–L2 level are more likely to develop DDD. This suggests that increased DSFT may be a contributing factor to DDD.

## 1. Introduction

Obesity has become a major public health issue worldwide in the recent decades. The number of adults in the Eastern Mediterranean region who are overweight or obese has increased dramatically from 15% to 21%, between 1980 and 2015 [1]. Therefore, if preventive measures are not taken, the global number of cases is anticipated to rapidly rise by the year 2030 [2]. Furthermore, obesity is a major risk factor for many chronic diseases, including diabetes, cardiovascular disease, lung diseases, and lumbar vertebral pathologies [3,4,5,6]. Thus, physicians must identify these comorbidities and their potential adverse outcomes. Being aware of the disorders that are strongly related to obesity is important for timely diagnosis and treatment, as well as for identifying individuals who would benefit the most from weight loss.

Degenerative disc disease (DDD) is a condition characterized by the breakdown of the intervertebral discs, which normally act as cushions between spinal vertebrae allowing for the efficient transfer of loads in multiple directions and maintaining flexibility [7]. While DDD is not usually life-threatening, it can be a source of chronic pain and discomfort for many people. As the disc degrades, it can cause a range of symptoms, such as back pain, stiffness, muscle spasms, and loss of mobility that eventually impact quality of life [8,9]. This condition is often associated with aging, but it can also be caused by genetic, metabolic, and other environmental factors [10].

Among the factors contributing to DDD, obesity has been suggested to be associated with the disease. Liuke and colleagues conducted a population-based magnetic resonance imaging (MRI) study to track the development of lumbar disc degeneration over 4 years [11]. They found that middle-aged Finn men with a body mass index (BMI) of 25 kg/m^2^ or higher were at an increased risk of developing DDD. According to a research-based MRI study conducted by Hangai et al., which involved 270 elderly Japanese individuals, the authors found that high BMI values were associated with an increased risk of developing disk degeneration [12]. A study conducted by Lee et al. suggests a potential link between herniated nucleus pulposus and the fat content of paraspinal muscles. The study involved 108 patients who underwent MRI scans of their spines due to lower back pain. Their findings revealed a significant association between a higher coronal 2D fat fraction of paraspinal muscles and the presence of herniated nucleus pulposus (HNP) [13]. Importantly, the studies mentioned earlier used BMI to determine someone’s body composition. Although it is a convenient method and accessible in clinical routine practice, this method has lower sensitivity to differentiate the distribution of muscle and fat in the body, and obesity-related health issues are linked to the way body fat is distributed. Therefore, an alternative quantitative reliable tool that can measure body fat distribution is needed.

MRI adiposity measures are promising tools to assess patients’ general health status [14,15]. It has been reported that visceral and subcutaneous fat measurements are superior to BMI in metabolic complications assessment [16]. A recent study conducted by Storz et al. found that visceral adipose tissue (VAT) measured from MRI is closely related to muscle fat infiltration and heart disease [17]. Furthermore, having excess fat in the liver measured by MRI has been linked to type 2 diabetes [17].

While previous studies have assessed the association between adipose tissue measurements and several pathological conditions, there is a lack of research with discordant findings investigating the relationship between the DSFT measured from MRI and DDD [15,18,19,20]. The conflicting findings from the aforementioned studies can be attributed to the different populations, adipose tissue measurement tools, measurement sites (lumbar spine level), and variable statistical methods used. These limitations emphasize the demand for further studies to explore the association between the DSFT measured from MRI and DDD.

To overcome the shortcomings of previous studies, this study investigated how the thickness of dorsal subcutaneous fat affects DDD at every lumbar vertebra, using MRI. It will also examine how other common potential factors, such as age and gender, could affect this relationship.

## 2. Methods

### 2.1. Study Population

This single-center, retrospective cross-sectional study was conducted at the MRI department of King Abdulaziz University Hospital, Jeddah, Saudi Arabia. A total of 424 participants, out of 755 individuals who received MRI lumbar spine examinations, were recruited between December 2021 and August 2022. Inclusion criteria were participants who had MRI lumbar spine study conducted at our institute and who were adequate to measure the thickness of back subcutaneous fat. Those who had unsuitable MRI images for measuring the thickness of back subcutaneous fat, surgery, trauma, metastatic lesions, and congenital anomalies were excluded from the study. Of 424 participants, 301 with DDD were chosen as the case group, and 123 without the disease were the control group. The Pfirrmann grading system, a well-established technique employed to evaluate the level of disc degeneration, was utilized for the evaluation of disc degeneration. This methodology takes into consideration various morphological and structural characteristics of the intervertebral discs, including disc height reduction, annular fissure, disc bulge, narrowing of the spinal canal, protrusion, and disc extrusion. Participants lacking any of these features were classified as the control group (without degenerative disc disease), whereas those displaying at least a single attribute constituted the case group (with degenerative disc disease). Data on demographic and clinical characteristics of the population such as age, gender, BMI, and the presence of DDD were retrieved from the radiology information system (RIS) and hospital information system (HIS).

### 2.2. Lumbar Magnetic Resonance Imaging

All patients have undergone a lumbosacral spine MRI scan on a high-field strength 3.0 Tesla machine (Magentom, Siemens, Malvern, PA, USA). The scan was performed while the patients were in a supine position using a multichannel phased array spine surface coil for optimal imaging. All images were acquired using the routine protocol: Sagittal spin-echo T2 (TR 3900 ms, TE 100 ms), Sagittal Stair T2 (TR 3000 ms, TE 53 ms), Sagittal spin-echo T1 (TR 620 ms, TE 10 ms), Axial spin-echo T2 (TR 5000 ms, TE 94 ms). The field of view was 320 × 320 mm^2^, with an acquisition matrix of 256 × 256 and a slice thickness of 3.5 mm.

### 2.3. Dorsal Subcutaneous Fat Thickness Measurements

**Figure 1:**
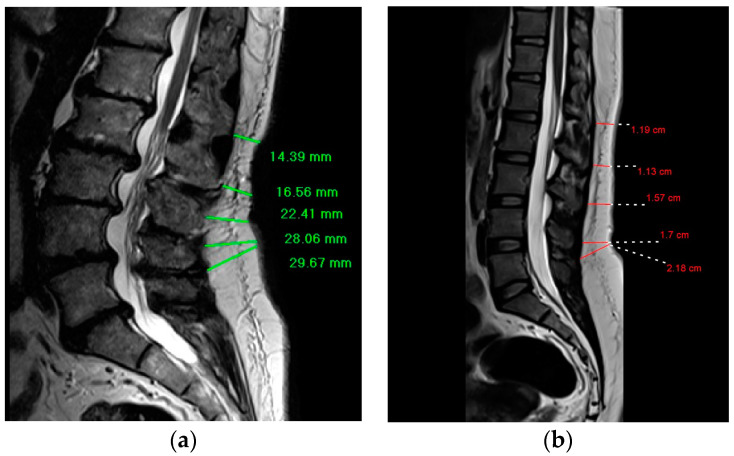
Demonstrates the way of the DSFT measurements for participants with degenerative disc disease and participants without the disease; (**a**) Mid-sagittal T2-weighted magnetic resonance image shows the measurement of the DSFT in a patient with worse degenerative disc disease at L1–L2 and L2–L3; (**b**) Mid-sagittal T2-weighted magnetic resonance image shows the measurement of the DSFT in a patient without the disease.

The DSFT in the current study represents the distance between the anterior aspect of dorsal subcutaneous fat and the back skin. Using length functions of MRI console, the DSFT of L1 to S1 intervertebral disc levels was measured in mid-sagittal spin-echo T2 weighted image where a line parallel to intervertebral disc space and extending from dorsal subcutaneous fat was drawn to measure the distance. (Figure 1) The measurements were taken by two radiologists with MRI experience of more than 10 years.

### 2.4. Statistical Analysis

Statistical analysis was performed by JASP software (0.16.3). Data were presented as frequencies and proportions if they had categories. The Shapiro–Wilk test and a histogram were used to check the normality of numerical variables, which were found to be not normally distributed. Therefore, the continuous variables were described as medians and interquartile ranges. To investigate the differences between the case and control groups in terms of population characteristics, the Chi-squared test (X^2^) was used for categorical variables. Because the continuous data did not follow a normal distribution, we adopted a non-parametric statistical test, namely the Mann–Whitney U test. This test was used to investigate the differences between the case and control groups in terms of population characteristics as well as the differences in the DSFT at all lumbar vertebral disc levels between males and females. The differences in the DSFT by gender were investigated to explore the hypothesis of women being more effective in storing fat subcutaneously compared to men. Doing so would help to reveal whether this fat layer provides a more helpful measure of obesity-related lumbar spine problems in females compared to males. A logistic regression model was built to explore the association between DDD and the thickness of dorsal subcutaneous fat at the L1–L2 level. A stratified logistic regression analysis by age and gender was also performed to explore the effect of these factors on the relationship. Odds ratios as well as 95% confidence intervals were computed to describe the differences between case and control groups. A value of *p* ≤ 0.05 in the analysis was deemed statistically significant. To assess how consistently different two observers measured the thickness of dorsal subcutaneous fat for each intervertebral disc level, we calculated inter-observer reliability using interclass correlation coefficients (ICCs) for all study populations (see Table 1). A two-way random model with absolute agreement was used. Scores were interpreted as poor (<0.40), fair (0.40–0.59), good (0.60–0.74), or excellent (0.75–1.00).

## 3. Results

The repeatability of the DSFT measurements (ICC) between the two radiologists was excellent, at 0.99, 0.99, 0.98, 0.99, and 0.99 for L1–L2. L2–L3, L3–L4, L4–L5, and L5–S1 levels, respectively (see Table 1).

The study includes 424 participants with a mean age of 49.59 years. More than half of the participants were male (58.5%) and older than 40 years (68.63%). The participants’ body mass indexes were 40.3% healthy, 36.2% overweight, 21.7% obese, and 1.7% underweight. The DSFT was measured for each lumbar vertebral body level and was found 1.47 cm, 1.47 cm, 1.87 cm, 2.32 cm, and 2.74 cm for L1–L2. L2–L3, L3–L4, L4–L5, and L5–S1 levels, respectively. Patients with DDD were older (78.6%) (*p* < 0.001), female (74.5%) (*p* = 0.05), and obese (72%) (*p* = 0.01). Compared to the group without the disease, those with DDD had significantly increased DSFT at the L1–L2 level (*p* = 0.01) (see Table 2).

Compared to males, females had a higher DSFT at the L4–L5 level (*p* < 0.001) and L5–S1 level (*p* < 0.001) (see Table 3). According to the logistic regression analysis, the odds of having DDD increased by 30% per one-millimeter increase in the DSFT at the L1–L2 level (*p* = 0.03). However, no relationship between subcutaneous fat thickness and disc degeneration was observed for the other levels (*p* ≥ 0.14) (see Table 4). When the logistic regression analysis was stratified by age and gender, a positive association between DDD and DSFT at L1–L2 level was observed among younger age group and female participants [OR _young_: 1.48; 95% CI (1.02–2.20); *p =* 0.04—OR _female_: 1.37; 95% CI (1–1.88); *p =* 0.05] (see Table 5).

## 4. Discussion

Obesity has been regarded as a risk factor for DDD; the more weight you gain, the greater the risk of having DDD you will have [11,12]. Thus, obesity should be quantified when DDD is assessed. Although anthropometric measures, such as BMI and waist circumference, are commonly used as indices of body fatness, these tools are limited by the poor sensitivity to discern the distribution of body muscle and fat. Therefore, the current study aims to explore the association between the DSFT measured from MRI and DDD. Findings from the current study show that the thickness of dorsal subcutaneous fat at the L1-L2 is significantly associated with DDD. Consistent with our findings, a recent study conducted by Ozcan-Eski and colleagues shows that there is a 12% higher odds of having DDD per one-millimeter increase in the DSFT at the L1–L2 level [20]. Moreover, Berikol et al. study reported that the DSFT of more than 8.45 mm among males and more than 9.4 mm among females is associated with DDD [19]. However, our work differs from these studies in that stratified logistic regression models by age and gender were considered. The use of those models revealed that the association of the DSFT with DDD is significantly pronounced among certain populations, namely younger and female groups.

In contrast to our findings, the studies by Yang et al. and Takatalo et al. reported no relationship between the DSFT and DDD. These studies, however, measured the thickness of dorsal subcutaneous fat of L4–L5 and L5–S1 levels only, which was found to have an insignificant relationship with the disease by previous studies and the current work [18,21].

Research revealed that subcutaneous fat thickness decreases with aging [22]. This decline with age means that older participants tend to have less DSFT, which potentially skews its importance in function toward younger populations in the current study. Given this age bias, our findings linking DDD to the DSFT in younger individuals should be treated with caution. A more balanced age distribution in future studies is crucial to confirm this relationship.

Prior studies supported the potential clinical utility of the DSFT at the L1–L2 level in the prognostication of several spinal disorders [20,23]. A recent study conducted by Ozcan-Eksi et al. reported that the DSFT measured from MRI and CT at LI–L2 level is significantly associated with DDD [20]. West et al.’s study found that the DSFT at the L1–L2 level can predict edema in the lumbosacral subcutaneous fat tissue [23]. These observations, along with our study findings, suggest that the thickness of the dorsal subcutaneous fat at the L1–L2 level can uniquely explain the lumbar spine pathologies induced by obesity compared to the measured fat thickness of other lumbar spine levels.

It is well established that adipose tissue distribution differs by gender; females are known to have an increased amount of subcutaneous fat, while males are characterized with more visceral adipose tissue [24]. Therefore, it was hypothesized that dorsal subcutaneous fat will be more informative about obesity-induced lumbar spine disorders among females. The measurement of the DSFT at L1–L2 level in the current study supports this hypothesis where each 1 mm increment in the DSFT at L1–L2 level increased the likelihood of having DDD in females by 37%. Additionally, the study by Takatalo et al. showed that each 1 mm increment in the abdominal and sagittal diameters, which both measure the visceral adipose tissues, increased the likelihood of having DDD in males by 67% and 40%, respectively [18].

The underlying exact mechanisms on how the thickness of subcutaneous fat at the L1–L2 level can cause DDD are complex and have yet to be explored. A potential explanation for the association between the thickness of subcutaneous fat at the L1–L2 level and DDD is that the increased thickness of fat at this level, particularly, may limit the ability of adipose tissue to recruit more adipose cells, which are found to be responsible for regulating body weight homeostasis [25]. Consequently, the excess fat could be ectopically deposited either in (1) the abdominal cavity, which may increase the mechanical load on the disc, causing a change in the disc structure as well as the biochemical components of the extracellular matrix; or in (2) the major blood vessels of the abdomen such as aorta, causing a reduced flow of blood to the disc (atherosclerosis), which eventually leads to disc degeneration and dehydration. Furthermore, our findings cannot ignore the role of hormonal factors in the association between subcutaneous fat and degenerative disease. According to the literature, adipose tissue releases pro-inflammatory molecules such as leptin and interleukins, which are associated with the degenerative process of joint cartilage [26].

It may be worth noting that females have thicker subcutaneous fat at the lower lumbar spine vertebrae than males, in accordance with the findings from the studies by Okan et al. and West et al. This could be due to the abundance of estrogen receptors in the subcutaneous fatty tissue of women compared to men, which have been proven to promote fat accumulation in the subcutaneous fat layer [27].

Apart from anthropometric measures, the DSFT can be quantified by different imaging methods including magnetic resonance imaging (MRI) and computed tomography. In the current study, the thickness of subcutaneous fat was measured by MRI. Compared to CT, this modality has no ionizing radiation and has two kinds of variable image contrast (T1 weighted image and T2 weighted image) that are easily capable of recognizing subcutaneous fat. Additionally, the subcutaneous fat measurements from MRI are more reproducible than CT [20].

It is estimated that close to 25% of the Saudi population is obese with a higher prevalence in females compared to males, putting them at a higher risk of developing degenerative disc disease [28]. Additionally, BMI, which is currently used as a proxy of the disease, has been found to weakly predict low back pain [16]. Therefore, exploring an alternative quantitative reliable screening method to predict DDD induced by obesity can lead to a more effective preventive strategy. The current study suggests a new potential way to improve the screening of DDD by measuring the dorsal subcutaneous fat at the L1–L2 level of Saudi women. If future studies with larger sample sizes and longer follow-up periods accounting for genetic, environmental, and lifestyle factors confirm our findings, this could lead to improved methods for screening and counseling women with obesity-related lumbar spine pathologies.

Although MRI offers excellent soft tissue differentiation and can measure subcutaneous fat thickness, its high cost compared to alternatives like ultrasound raises concerns about its applicability for this purpose. Typically, medical insurance covers treatments and procedures deemed medically necessary based on evidence-based guidelines. As spine MRI for measuring the DSFT is not a standard practice for obesity assessment in Saudi Arabia, and MRI is usually reserved for specific medical indications due to its cost and resource constraints, employing it solely for fat thickness measurement, particularly in an obesity context, is likely deemed an unnecessarily expensive approach.

To our knowledge, this is the first study that assesses the relationship between the thickness of subcutaneous fat and DDD in the Saudi population. Studies on the relationship between the thickness of subcutaneous fat and degenerative disc disease to date have been conducted on populations with different ethnicities and lifestyles from the Saudi population, making it difficult to compare their findings to the current study [18,19,20,21]. Thus, our work provides a foundation for future research in Saudi Arabia by establishing a starting point for comparison. In addition to that, the thickness of subcutaneous fat measurements was from MRI and conducted by two experienced radiologists with excellent agreement on the fat measurements, suggesting the reliability of the measurements taken. Furthermore, important confounders such as age and gender were considered in the logistic regression models. Such factors have been shown to have an impact on both DDD and subcutaneous fat body distribution and were rarely deemed in the prior studies that investigated the relationship between the thickness of subcutaneous fat and DDD.

This study also has some limitations that should be acknowledged. A possible drawback of the current research is that it is based on a single center, which potentially restricts the generalizability of findings. The retrospective nature of the study also does not capture the impact of all potential confounders including genetic, environmental, and lifestyle factors that could bias our findings. Additionally, the study was unable to investigate the relationship between the severity of DDD and DSFT since the data on the severity of DDD were very limited. Therefore, larger multiethnic prospective studies accounting for the previously mentioned limitations are needed to validate our results.

## 5. Conclusions

The findings show that female and younger individuals with higher subcutaneous fat thickness at the L1–L2 level have increased the odds of developing DDD, indicating that increased dorsal subcutaneous fat may be a contributing factor to lumbar disc degeneration. A large longitudinal study would help learn more about the relationship between dorsal subcutaneous fat and lumbar disc degeneration.

## Figures and Tables

**Table 1:** The interclass correlation coefficient (ICC) of the DSFT measurements for each vertebral body level.

Vertebral Body Level	Point Estimate	Lower 95% Cl	Upper 95% Cl
L1–L2	0.990	0.998	0.992
L2–L3	0.991	0.990	0.993
L3–L4	0.986	0.983	0.988
L4–L5	0.993	0.992	0.994
L5–S1	0.995	0.994	0.996

**Table 2:** Distribution of degenerative disc disease status by population characteristics.

Population Characteristics		All Patients	Patients with DDD	Patients without DDD	*p*-Value
Age n (%)	Less than 40 years	133 (31.36%)	72 (54.1%)	61 (45.8%)	<0.001
	More than or equal to 40 years	291 (68.63%)	229 (78.6%)	62(21.3%)	
Gender n (%)	Male	248 (58.5%)	116 (65.9%)	60 (34%)	0.05
	Female	176 (41.5%)	185 (74.5%)	63(25.4%)	
BMI n (%)	Healthy	139 (40.3)	89 (64%)	50 (35.9%)	0.01
	Underweight	6 (1.7%)	2 (33.3%)	4 (66.6%)	
	Overweight	125 (36.2%)	98 (78.4%)	27 (21.6%)	
	Obese	75 (21.7%)	54 (72%)	21 (28%)	
DSFT at L1–L2 level: median (IQR) cm		1.47 (0.85–2.22)	1.52 (0.9–2.29)	1.28 (0.69–1.99)	0.01
DSFT at L2–L3 level: median (IQR) cm		1.47 (0.90–2.19)	1.49 (0.93–2.23)	1.45 (0.76–2.16)	0.28
DSFT at L3–L4 level: median (IQR) cm		1.87 (1.23–2.81)	1.87 (1.31–2.83)	1.75 (1.08–2.75)	0.2
DSFT at L4–L5 level: median (IQR) cm		2.32 (1.62–3.41)	2.35 (1.69–3.46)	2.29 (1.38–3.21)	0.15
DSFT at L5–S1 level: median (IQR) cm		2.74 (1.87–3.72)	2.86 (1.98–3.72)	2.70 (1.64–3.73)	0.16

Abbreviations: DDD: disc degenerative disease; BMI: body mass index; n: number of participants; IQR: interquartile range; DSFT: dorsal subcutaneous fat thickness.

**Table 3:** Differences in the DSFT by gender.

The Level of Subcutaneous Fat Thickness Measurement	Male	Female	*p*-Value
DSFT at L1–L2 level: median (IQR) cm	1.52 (0.89–2.1)	1.38 (0.80–2.32)	0.9
DSFT at L2–L3 level: median (IQR) cm	1.53 (0.89–2.12)	1.45 (0.90–2.33)	0.7
DSFT at L3–L4 level: median (IQR) cm	1.81 (1.12–2.57)	1.94 (1.29–2.98)	0.03
DSFT at L4–L5 level: median (IQR) cm	2.11 (1.35–3.03)	2.6 (1.68–3.66)	<0.001
DSFT at L5–S1 level: median (IQR) cm	2.43 (1.62–3.38)	3 (2.04–3.89)	<0.001

Abbreviations: IQR: interquartile range; DSFT: dorsal subcutaneous fat thickness.

**Table 4:** The odds ratio for having degenerative disc disease concerning the DSFT (at L1–L2 level).

The Level of Subcutaneous Fat Thickness Measurement	Presence of Degenerative Disc Disease Crude OR (95% CI)	*p*-Value
DSFT (at L1–L2 level)	1.30 (1.02–1.64)	0.03
DSFT (at L2–L3 level)	1.09 (0.87–1.36)	0.43
DSFT (at L3–L4 level)	1.10 (0.91–1.32)	0.31
DSFT (at L4–L5 level)	1.12 (0.95–1.33)	0.16
DSFT (at L5–S1 level)	1.12 (0.95–1.32)	0.14

Abbreviations: OR: odds ratio; (95% CI): 95% confidence interval; DSFT: dorsal subcutaneous fat thickness.

**Table 5:** The odds ratios for having degenerative disc disease concerning the DSFT (at the L1–L2 level) are stratified by age and gender.

The Level of Subcutaneous Fat Thickness Measurement			Presence of Degenerative Disc Disease OR (95% CI)	*p*-Value
DSFT (at L1–L2 level)	Age	Less than 40 years	1.48 (1.02–2.20)	0.04
		More than or equal to 40 years	1.11 (0.82–1.52)	0.47
	Gender	Female	1.37 (1–1.882)	0.05
		Male	1.2 (0.830–1.745)	0.32

Abbreviations: OR: odds ratio; (95% CI): 95% confidence interval; DSFT: dorsal subcutaneous fat thickness.

## Data Availability

The original contributions presented in the study are included in the article, further inquiries can be directed to the corresponding author.
